# Bolus of Hair Causing Bowel Obstruction and Requiring Laparotomy

**Published:** 1896-11

**Authors:** 


**Bolus of Hair Causing Bowel Obstruction and Requiring Laparotomy.**—Dr. J. S. Wellford exhibited a bolus of hair, about an inch and a half in diameter and three inches long, removed from the small intestine of a girl aged fourteen. There were appearances of beginning menstruation. Pain appeared first in the right iliac region, but soon spread over the whole abdomen, and was intense. There was no fever or increase of pulse, and bowel operations were small. Dr. Long was called in consultation and thought it a ruptured appendicitis. Upon operation, this bolus of hair was found completely occluding the lumen of the intestine. There was violent vomiting, which, just before the operation, became stercoraceous.

Dr. Virginius W. Harrison reported the case of a boy, aged three years, who had been in the habit of eating wall paper, and finally passed balls of it. Constant surveillance was necessary to keep him from it.

Dr. Upshur reported a case of *puerperal mania.* The patient had been afflicted for six or eight years with chronic diarrhœa. Previous to her marriage she had been a shirt maker. During the spasms, she passed a large ball composed of cotton threads, which it is supposed she swallowed in biting off threads to thread her needles. The interesting point here is not the obstruction, but chronic diarrhœa which resulted. After recovery from mania, she was put on treatment for the diarrhœa and is now entirely well.—*Richmond Acad. Med.*

